# Immunomodulatory and anti-inflammatory effects of *Phellinus linteus* mycelium

**DOI:** 10.1186/s12906-021-03441-9

**Published:** 2021-10-26

**Authors:** Mi-Rae Shin, Ji Hye Lee, Jin A Lee, Min Ju Kim, Hae-Jin Park, Byeong Wook Park, Seung Bo Seo, Seong-Soo Roh

**Affiliations:** 1 Department of Herbology, College of Korean Medicine, Daegu Haany University, 136, Shinchendong–ro, Suseong-gu, Deagu, 42158 Republic of Korea; 2 College of Korean Medicine, Semyung University, 65, Semyung-Ro, Jecheon, Chungbuk 27136 Republic of Korea; 3 DHU Bio Convergence Testing Center, 1, Hanuidae-ro, Gyeongsan-si, Gyeongsangbuk-do 38610 Republic of Korea; 4 Hankook Shinyak Pharm. Co. Ltd, 39-83 Zhongshan-gil, Yangchon-myeon, Nonsan-si, Chungcheongnam-do 33023 Republic of Korea

**Keywords:** *Phellinus linteus* mycelium, RAW264.7 cells, Immunomodulatory, Anti-inflammatory, Th1 response

## Abstract

**Background:**

The present study extensively aimed to evaluate the underlying mechanism of the immunomodulatory and anti-inflammatory effects of *Phellinus linteus* mycelium (PLM).

**Methods:**

To assess whether PLM influences the production of markers related to inflammation, Lipopolysaccharide (LPS)-stimulated RAW264.7 cells were treated with PLM (50, 100, 200, and 500 μg/mL). Splenocyte, thymus, peritoneal exudate cells (PEC), and peripheral blood mononuclear cells (PBMC) were isolated from the Balb/c mice treated with Korean red ginseng or PLM once a day for 5 weeks. Moreover, all mice except normal mice were stimulated with 10% proteose peptone (PP) treated 3 days before the sacrifice and 2% starch treated 2 days before the sacrifice. Subsequently, the cytotropic substance was evaluated by using flow cytometry analysis and ELISA assay.

**Results:**

PLM200 treatment significantly suppressed the production of nitric oxide (NO) and prostaglandin E2 (PGE2) and inhibited the release of proinflammatory cytokines such as interleukin (IL)-6, IL-1β, and tumor necrosis factor (TNF)-α dose-dependently in the LPS-stimulated RAW264.7 cells. PLM200 supplementation showed a significant increase in IL-2, IL-12, and interferon (IFN)-γ production and upregulated the ratio of IFN-γ (T-helper type 1, Th1) to IL-4 (T-helper type 2, Th2) in splenocytes. After PLM200 treatment, the significant elevation of CD4^+^CD25^+^, CD4^+^&CD8^+^, and CD4^+^CD69^+^ treatment were detected in thymus. Moreover, CD4^+^ and CD4^+^CD69^+^ in PBMC and CD69^+^ in PEC were also shown in a significant increase.

**Conclusions:**

Taken together, these results showed an immunomodulatory effect of PLM about an elevated INF-γ/IL4 ratio, as an index of Th1/Th2, as well as the anti-inflammatory effect in the LPS-stimulated RAW264.7 cells. Therefore, our findings demonstrate that PLM possesses immunostimulatory and anti-inflammatory effects.

## Background

Inflammation plays a decisive role in the control of the infestation of external pathogens. Traditionally, innate immunity has been described as triggering generic and nonspecific immune responses rapidly. When pathogens reach the initial barriers of the skin or a mucosal surface, innate defense mechanisms are encountered and then an inflammatory response is quickly initiated [1]. Some of the most potent soluble antimicrobial factors encountered include complement, lysozymes, dermcidin, defensins, cathelicidins, mucins, and lectins [2, 3]. Several of these mediators are pluripotent and perform antimicrobial functions. That is, they amplify the inflammatory response triggered in resident sentinel immune cells upon pathogen sensing [1, 4]. Within minutes to hours of detection of alarm signals, a “heightened alert” inflammatory transcriptional program stimulates in sentinel innate immune cells including tissue macrophages and dendritic cells. This program causes by the generation of an antipathogen state and the production of numerous inflammatory cytokines and chemokines [5].

A representative characteristic of inflammation is the migration of polymorphonuclear neutrophils (PMNs) from peripheral blood to the inflammatory site [6]. As a first-line defense, PMNs promptly migrate into the inflamed site to kill the invading pathogens. Once PMNs have completed the phagocytosis, an orderly elimination of PMNs by macrophages is intrinsic for the resolution of inflammation [7, 8]. Macrophages have been described as major regulators of inflammatory responses. In response to various signals, macrophages can divide into two distinct phenotypes (M1 and M2): The M1-like macrophages are characterized by high levels of proinflammatory cytokines, strong tumoricidal activity, high production of reactive nitrogen, and oxygen intermediates. Whereas, M2-like macrophages are considered to be involved in parasite control, wound healing, immune regulation as well as tumor promotion [9, 10]. As indicated above, M1-like macrophages produce proinflammatory cytokines such as TNF-α, type I interferon (IFN), IL-1β, IL-6, IL-12, and several chemokines and induce Th1 response activation under acute inflammatory responses. M2-like macrophages produce anti-inflammatory cytokines, such as IL-4, IL-13, IL-10, and induce Th2 response activation [11]. If the immune response fails to effectively control the pathogen, it leads to clinical disease.

Medicinal fungi are well-known for producing diverse bioactive metabolites including antibiotics and anti-cancer drugs [12, 13]. A number of medical metabolites have been reported from various edible fungi. Traditionally, *Phellinus linteus* mycelium (PLM) has been used as medicine or healthy food for the treatment of several diseases, such as gastroenteric disorder, various cancers, lymphatic diseases [14]. Accumulating evidence shows PLM exerts a wide variety of medicinal potencies such as antioxidant, anti-inflammatory, anti-viral, cytotoxic, and anti-diabetic effects [15, 16]. We previously reported the protective effects of PLM on the development of osteoarthritis after monosodium iodoacetate (MIA) injection [17]. Herein, PLM exerted an outstanding chondroprotective effect through the suppression of both ROS overproduction and inflammation. Moreover, many studies have shown that β-glucans have potential immunostimulatory properties [18, 19].

Based on the previous studies, we hypothesized that bioactive compounds of PLM might alter several biological systems, including the immune system. Therefore, this study extensively aimed to evaluate both the anti-inflammatory effect in RAW264.7 cells and the immunomodulatory activities of PLM in PP and starch-treated Balb/c mice.

## Methods

### Preparation of PLM

PLM was obtained as a dried mycelium from Hankook Shinyak Corp. (Nonsan-si, Korea) and Green-lipped mussel powder was obtained from McFarlane Marketing (Aust) Pty Ltd. (Melbourne, Australia). *Phellinus linteus* mycelium was inoculated under optimal culture conditions (at 30 °C and pH 4–5) for 14 days on yeast malt extract glucose (YMG) agar. The extract condition was optimized for extraction temperature (100 °C), extraction time (24 h), solvent amount (10 times purified water), and extraction frequency (1st). A 50-ton large-scale incubator was used to produce 7 g/L dry mycelium in 4 days after mass production of mushroom mycelium. The produced PLM was analyzed the nutritional components in accordance with the method of a food revolution at Korea Health Supplement Institute (Seongnam, Korea) [20]. The composition of PLM showed Table 1.

**Table 1:** Analysis of Nutritional Compositions of PLM

Calorie (Kcal/100 g)	Carbohydrate (%)	Protein (%)	Fat (%)	Sodium (mg/ 100 g)	Sugar (mg/ g)	Saturated fatty acid (g/ 100 g)	Trans fat (g/ 100 g)	Cholesterol (mg/ 100 g)
346.4	68.9	15.8	0.9	71.6	128.3	0.1	0	0

### β -glucan measurement of PLM

β-glucan content in PLM was performed using K-YBGL kit (Megazyme, Ireland) as the mushrooms and yeast β-glucan assay procedure. Extraction, laboratory analysis, and calculation were performed in accordance with the manufacturer’s instructions. The content of β-glucan was calculated using the following formula:

$$\upbeta -\mathrm{glucan}\ \mathrm{content}\ \left(\%\right)=\left(\mathrm{Total}\ \mathrm{glucan}\ \mathrm{content}\right)-\left(\mathrm{content}\ \mathrm{excluding}\ \upbeta -\mathrm{glucan}\right)$$

Consequently, β-glucan content of PLM was 14.16 ± 6.27%.

### Cell culture

RAW264.7 cells were purchased from the American Type Culture Collection (ATCC, Manassas, VA, USA). Cells were cultured using Dulbecco’s modified Eagle’s medium (DMEM, Gibco Inc., Grand Island, NY, USA) containing 5.5% heat-inactivated fetal bovine serum (FBS, Gibco Inc.), penicillin (100 U/mL), and streptomycin (100 μg/mL) and maintained in atmosphere of 95% humidity and 5% CO_2_ at 37 °C incubator. PLM (50, 100, 200, and 500 μg/mL) was dissolved in 100% dimethyl sulfoxide (DMSO) and each sample was diluted to a final content of 0.1% DMSO. And then cells were incubated for 24 h.

### Cell viability

Cell viability assays were measured to confirm the cytotoxicity of PLM using 3-(4,5-dimethylthiazol-2-yl) − 2,5-diphenyltetrazolium bromide (MTT) obtained from Sigma-Aldrich Chemical Co.(St. Louis, MO, USA). MTT dissolved in serum-free DMEM (final concentration of 0.5 mg/mL) and 100 μL of this solution was treated to cell cultures for 4 h in 96-well culture plates. One hundred microliter of DMSO is added to dissolve the formazan crystals. To measure cell viability, absorbance was measured at 570 nm using a microplate reader (SpectraMax M2, Molecular Devices, USA).

### NO production and pro-inflammatory cytokine assay

RAW264.7 cells (at a density of 2.5 × 10^5^ cells/well) were treated with PLM for 1 h and then exposed to 1 μg/mL LPS. After 24 h, the nitrite level to evaluate NO formation was measured using Griess Reaction assay. One hundred microliter aliquots were mixed with an equal volume of Griess reagent and incubated at room temperature for 10 min. The absorbance at 550 nm was measured in a microplate reader (Bio-Rad, Hercules, CA, USA) as a measurement of NO production. The levels of PGE2, TNF-α, IL-1β, and IL-6 were evaluated using ELISA kits obtained from R&D Systems (Minneapolis, MN, USA) according to the manufacturer’s protocol. Moreover, serum IL-2, IL-12, IL-4, IL-10, and IFN-γ were measured using the mouse inflammation kit CBA (BD Bioscience, San Diego, CA) according to the manufacturer’s protocol.

### Mice and activation of macrophage

Seven-week-old male Balb/c mice were obtained from OrientBio (Seongnam, South Korea). The animal experiment was conducted in accordance with the Guide for the Care and Use of Laboratory Animals by the National Institutes of Health (NIH) and the current study is approved by the committee for animal welfare at Daegu Haany University (Permit Number: DHU2019–055). The mice were maintained at 23 ± 2 °C and controlled with a 12 h light/dark cycle and humidity (55 ± 5%). Mice were fed a commercial diet (NIH-41, Zeigler Bros, Inc., USA) and water ad libitum for adaptation (1 week) before beginning the experiment. Mice are randomly arranged in descending order of weight and assigned into five groups of equal number (*n* = 7) without statistical significance among the groups. The normal group was administrated saline using a stomach tube, while the other groups were orally administered red ginseng 100 mg/kg or PLM 50, 100, and 200 mg/kg using a stomach tube once daily for 5 weeks. The experimental mice were orally administered once daily for 5 weeks. 10% pentose peptone was treated 3 days before the sacrifice and 2% starch was treated 2 days before the sacrifice. The body weight (g) on the day of sacrifice was the Control group (22.28 ± 0.40), the KRG100 group (24.21 ± 0.30), the PLM50 group (23.53 ± 0.41), the PLM100 group (23.58 ± 0.52), and the PLM200 group (23.62 ± 0.26), and there was no significant difference between groups. Mice were sacrificed by inhalation anesthesia using isoflurane (Troikaa Pharmaceuticals Ltd., Gujarat, India). When the toes of anesthetized mice were irritated and did not show movement, it was judged as unconscious. Then, blood was collected by cardiac puncture and centrifuged at 3000 rpm for approximately 20 min at 4 °C. The collected serum was stored at − 80 °C until analysis.

### Sample collection and PBMCs preparation

Peripheral blood mononuclear cells (PBMCs) were obtained from heparinized blood samples via Percoll density-gradient centrifugation. Single-cell suspensions from the thymus and abdominal cavity were isolated in RPMI-1640 medium supplemented with 100 U/mL penicillin, 100 μg/mL streptomycin, 2 mM L-glutamine, 20 mM HEPES, 50 μM 2-mercaptoethanol, and 2% FBS. Briefly, the thymus was minced and incubated in PBS containing 1 mg/mL collagenase IV (Sigma) for 40 min at 37 °C. The filtered cell suspensions were centrifuged at 450×g for 20 min. Between 2 × 10^3^ and 4 × 10^3^ cells (100 μL) were spun onto glass slides via centrifuge at 400×g for 4 min using a cytospin centrifuge (Cellspin; Hanil, Seoul, Korea), and then stained with Diff-Quick Stain Set solution (Baxter Healthcare; Miami, FL, USA).

### FACS

The cells were stained with highly specific antibodies for flow cytometric analysis (FACS). Cells from the thymus, peritoneal exudate, and PBMCs were stained using the indicated antibodies with staining solution (PBS containing 1% FBS and 0.01% NaN_3_) for 10 min on ice. Next, the stained cells were evaluated by two-color flow cytometry on a FACSCalibur Setup using CellQuest Pro software (BD Biosciences; Mountain View, CA, USA). All antibodies for FACS, including anti-CD4^+^CD25^+^, anti-CD4^+^, anti-CD8^+^, anti-CD69^+^, and anti-CD4^+^CD69^+^ were obtained from BD PharMingen (San Diego, CA, USA). The number of absolute cells was showed as the percentage value of the total cell number.

### Statistical analysis

These experimental results are expressed as the mean ± standard error of the mean (SEM). In addition, they analyzed using one-way analysis of variance (ANOVA) followed by the Least-significant differences (LSD) test for multiple comparisons or unpaired Student’s t-tests when comparing the two groups. All data analyses were performed using the SPSS version 25.0 software (SPSS Inc., Chicago, IL, USA). Values of *P* < 0.05 were considered significant.

## Results

### Effects of PLM on cytotoxicity, NO, PGE2_,_ TNF-α, IL-6, and IL-1β productions in RAW264.7 cells

**Fig. 1:**
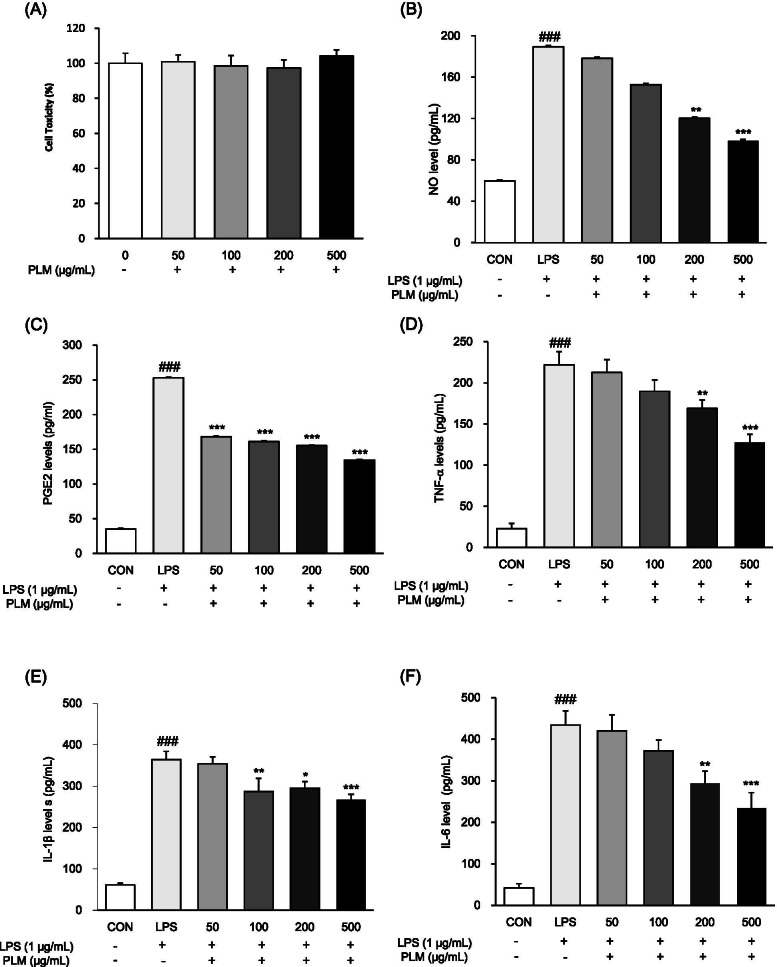
Effect of PLM on cytotoxicity, NO, PGE2_,_ TNF-α, IL-1β, and IL-6 productions in LPS-stimulated RAW264.7 cells. Cells were pretreated with PLM (0, 50, 100, 200, 500 μg/mL) for 2 h and then with LPS (0.5 μg/mL) for 24 h. **A** Cell viability was measured with an MTT assay. **B** Collected supernatants were reacted with Griess reagent, and the absorbance was measured at 540 nm. The supernatants were collected and subjected to ELISA for (**C**) PGE2, **D** TNF-α, **E** IL-1β, and **F** IL-6. The values are expressed as the mean ± SEM (*n* = 5). ^**###**^*p* < 0.001 vs. control cells, *p < 0.05; **p < 0.01, ***p < 0.001 vs. LPS-treated cells

To assess whether PLM influences the production of markers related to inflammation, LPS-stimulated RAW264.7 cells were treated with PLM. PLM did not show any considerable cytotoxicity in RAW264.7 cells (Fig. 1A). The effect of PLM on NO production was measured in LPS-treated RAW264.7 cells. As shown in Fig. 1B, PLM significantly decreased NO production at doses of 100 (19.3%), 200 (36.5%), and 500 (47.9%) μg/mL. We also investigated the inhibitory effect of PLM on PGE2, IL-6, IL-1β, and TNF-α generation in LPS-treated RAW264.7 cells. LPS significantly increased the levels of PGE2, TNF-α, IL-6, and IL-1β and whereas PLM significantly decreased the LPS-induced PGE2 (33.5, 36.3, 38.6, 46.8%, resp.) in a dose-dependent manner. Moreover, the levels of TNF-α (23.9, 42.6%), IL-6 (19.0, 26.7%), and IL-1β (32.2, 46.0%) production both 200 and 500 μg/mL significantly decreased (Fig. 1C–F).

### Effects of PLM on cytokine production in splenocytes

**Fig. 2:**
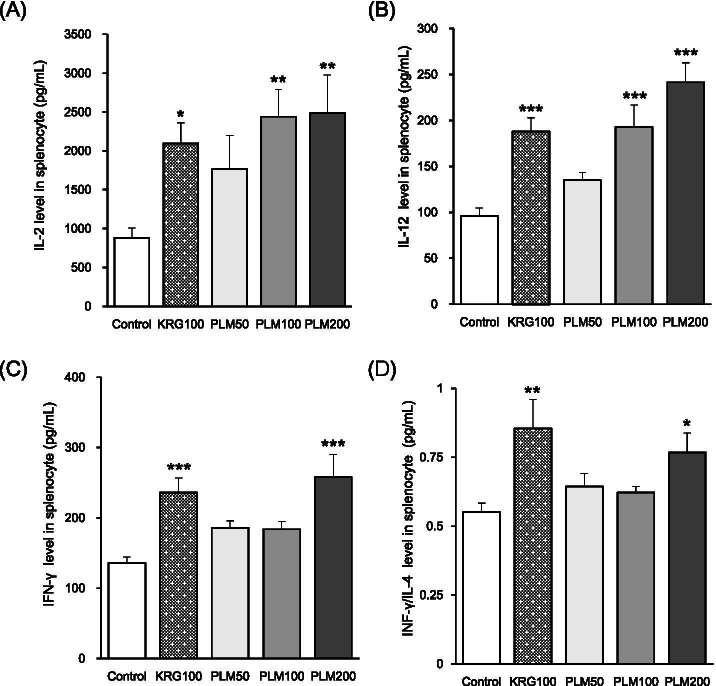
Effects of PLM on cytokine production in splenocytes. Mice were pretreated with KRG (100 mg/mL) or PLM (50, 100, and 200 mg/mL) orally for 5 weeks. Moreover, all mice except normal mice were stimulated with 10% PP treated 3 days before the sacrifice and 2% starch treated 2 days before the sacrifice. The splenocytes were collected and subjected to an ELISA kit for (**A**) IL-2, **B** IL-12, **C** IFN-γ and calculated (**D**) IFN-γ/IL-4 ratio. The values are expressed as the mean ± SEM (*n* = 7). *p < 0.05, **p < 0.01, ***p < 0.001 vs. Control mice

PLM (50, 100, and 200) treatment did not show any meaningful cytotoxicity in splenocytes. All drug-treated groups increased IL-2, IL-12, and IFN-γ production by splenocytes compared to PP and starch-stimulated mice (control) (Fig.2A, B, and C). Especially, three groups (KRG100, PLM100, and PLM200) showed a significant increase in all of three cytokines above. Moreover, the ratio of IFN-γ /IL-4 production was significantly higher than the control group (Fig. 2D). Compared with the control group, PLM200 significantly increased the ratio of IFN-γ /IL-4 production by 39.14%. These results strongly suggest that PLM is a potent immune-stimulating extract.

### Effects of PLM on total cell number in the thymus, PEC, and PBMC

**Fig. 3:**
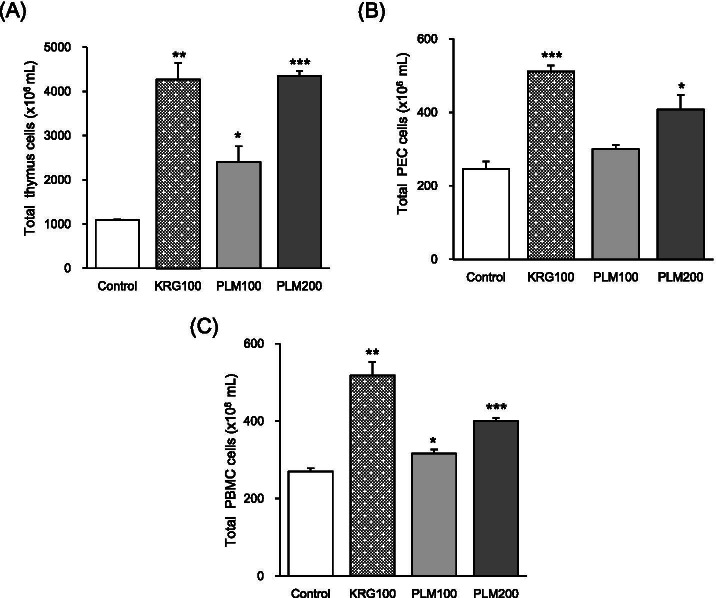
Effects of PLM on total cell number in the thymus, PEC, and PBMC. Mice were pretreated with KRG (100 mg/mL) or PLM (100, and 200 mg/mL) orally for 5 weeks. Moreover, all mice except normal mice were stimulated with 10% PP treated 3 days before the sacrifice and 2% starch treated 2 days before the sacrifice. Total cell numbers were counted in the thymus, PEC, and PBMC. **A** Thymus, **B** PEC, **C** PBMC. The values are expressed as the mean ± SEM (*n* = 3). *p < 0.05, **p < 0.01, ***p < 0.001 vs. Control mice

As shown in Fig. 3, total cell numbers were measured in the thymus, PEC, and PBMC. There are 1090 ± 17.32 (× x10^6^) cells in the control group and the numbers were significantly increased to 4270 ± 375.28 (× 10^6^), 2400 ± 357.96 (× 10^6^), and 4350 ± 109.7 (× 10^6^) in the KRG100, PLM100, and PLM200, respectively. In PEC, there are 246.5 ± 19.34 (× 10^6^) cells in the control group and the numbers were significantly increased to 511.5 ± 16.45 (× 10^6^) and 408.0 ± 39.26 (× 10^6^) in the KRG100 and PLM200, respectively. Besides, PLM100 didn’t show significance. In PBMC, there are 270.5 ± 7.79 (× 10^6^) cells in the control group and the numbers were significantly increased to 518.0 ± 34.64 (× 10^6^), 316.5 ± 10.10 (× 10^6^), and 400.0 ± 8.66 (× 10^6^) in the KRG100, PLM100, and PLM200, respectively. These results suggest that the increase of total cell numbers of tissue or cells associated with immune response leads to a strong immune activation.

### Effects of PLM on immune cell subtype in the thymus

**Fig. 4:**
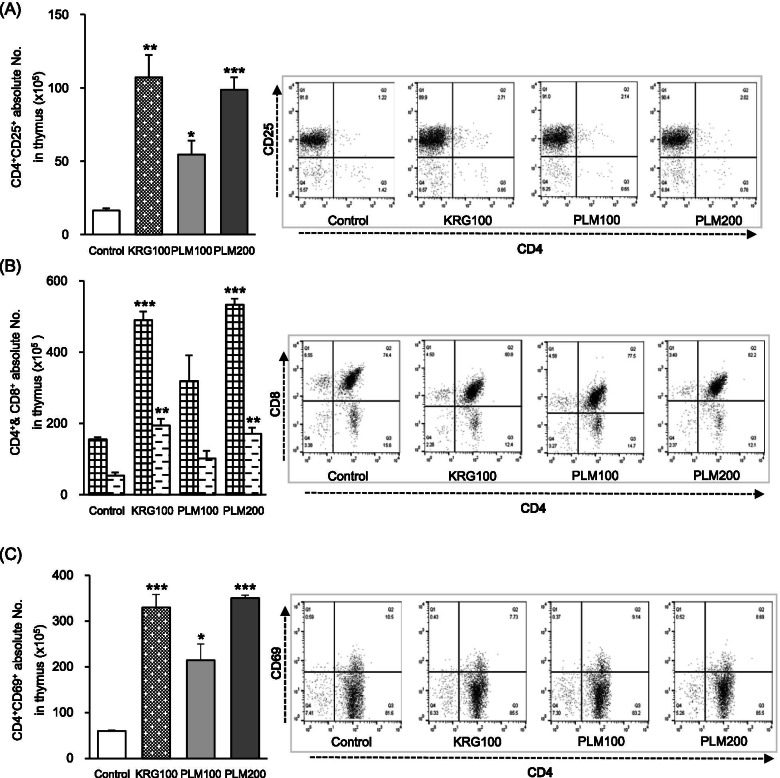
Effects of PLM on immune cells subtype in the thymus. Mice were pretreated with KRG (100 mg/mL) or PLM (100, and 200 mg/mL) orally for 5 weeks. Moreover, all mice except normal mice were stimulated with 10% PP treated 3 days before the sacrifice and 2% starch treated 2 days before the sacrifice. The thymus was collected and measured through fluorescent antibody cell sorting (FACS). **A** CD4^+^CD25^+^ absolute numbers, **B** CD4^+^& CD8^+^ absolute numbers, and **C** CD4^+^CD69^+^ absolute numbers. The values are expressed as the mean ± SEM (*n* = 3). *p < 0.05, **p < 0.01, ***p < 0.001 vs. Control mice

Analysis of the expression of immune cell subtypes was performed using the flow cytometry technique in the thymus. As shown in Fig. 4, an absolute number of CD4+CD25+ cells from the thymus were significantly elevated (PLM100; 3.3-fold, PLM200 6.1-fold). Moreover, PLM100 showed an increased tendency without a significance in an absolute number of CD4+&CD8+ cells. Besides, PLM200 significantly increased an absolute number of CD4+&CD8+ cells. Furthermore, the absolute number of CD4+CD69+ cells were significantly elevated (PLM100; 3.5-fold, PLM200 5.8-fold). Especially, PLM200 is 63.45% higher than PLM100. These results clearly indicate that PLM is potent as immune-boosting material because it caused the activation of T subtypes in the thymus.

### Effects of PLM on immune cell subtype in PEC and PBMC

**Fig. 5:**
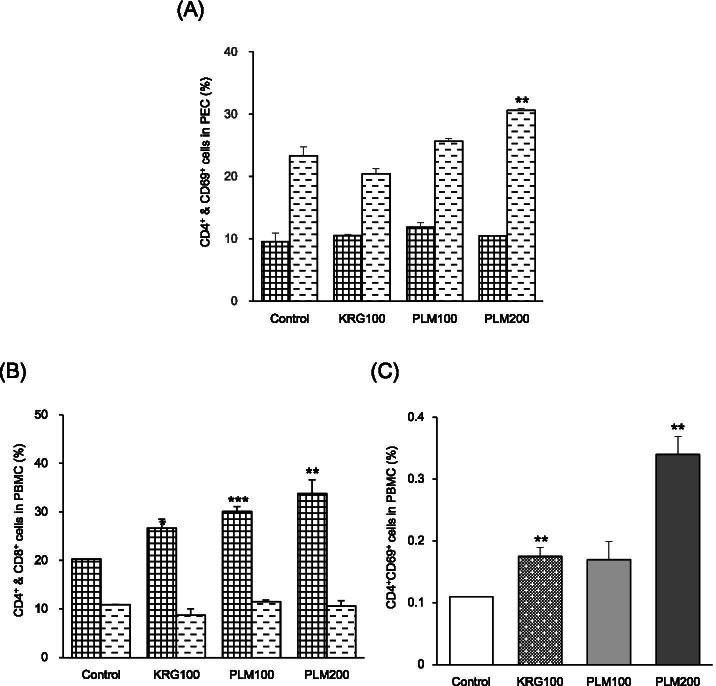
Effects of PLM on immune cells subtype in PEC and PBMC. Mice were pretreated with KRG (100 mg/mL) or PLM (100, and 200 mg/mL) orally for 5 weeks. Moreover, all mice except normal mice were stimulated with 10% PP treated 3 days before the sacrifice and 2% starch treated 2 days before the sacrifice. The PBMC and PEC were collected and counted percentage (%). **A** CD4^+^ & CD69^+^ cells in PEC, **B** CD4^+^CD69^+^ cells in PBMC, **C** CD4^+^ & CD8^+^ cells in PBMC, The values are expressed as the mean ± SEM (*n* = 3). **p < 0.01, ***p < 0.001 vs. Control mice

Analysis of the expression of immune cell subtypes was performed using the flow cytometry technique in PEC and PBMC. As shown in Fig. 5, CD4^+^&CD8^+^ cells (%) in PBMC showed a significant elevation at only CD4^+^ (PLM100; 48.3%, PLM200 66.5%). Especially, PLM200 is 12.3% higher than PLM100. Moreover, PLM100 showed an increasing tendency without significance in CD4^+^CD69^+^ cells (%) in PBMC. Besides, PLM200 significantly increased in CD4^+^CD69^+^ cells (%). Furthermore, CD4^+^CD69^+^ cells (%) in PEC showed a significant elevation at only CD69^+^ (PLM200 31.26%). Especially, PLM200 is 63.45% higher than PLM100. These results suggest that PLM is potent as immune-boosting material because it caused the activation of T cells subtypes in PBMC and PEC.

## Discussion

Many researchers have focused on immune therapies as better strategies to treat and manage cancer. Immunomodulation in cancer therapy closely involves immune cells that are extensively involved as antigen presenting cells (APCs), suppression of inflammation, activation of T lymphocytes, production of various cytokines, and direct cytotoxicity to cancer cells [21]. Therefore, materials with immunomodulatory activity as opposed to conventional therapies including chemotherapy, radiotherapy, and surgery that lead to immunosuppression are of considerable value in cancer immunotherapy. Extracts or mycelium based on fungi, lichens, and algae have elicited great interest among researchers due to their immunomodulatory and anticancer potential [22]. In this study, we confirmed a novel function of *Phellinus linteus* mycelium (PLM) as a potent therapeutic modulator and elucidated the underlying mechanisms because the immunomodulation potential of its polysaccharides has not been fully exploited.

Inflammation is a cascade reaction involving numerous inflammatory mediators [23]. The inflammatory process is generally characterized by the recruitment of leukocytes and macrophages. Lipopolysaccharide (LPS) quickly activates macrophages and stimulates the secretion of pro-inflammatory cytokines such as IL-1β, IL-6, and TNF-α as well as inflammatory mediators, such as NO and PGE2 [24]. The cytotoxicity of PLM (50, 100, 200, and 500 μg/mL) was measured by MTT assay. PLM did not show a cytotoxic effect at up to 500 μg/mL compared with untreated control cells (Fig. 1). The inhibitory effect of PLM on NO and Prostaglandin E2 (PGE2) production was analyzed in LPS-induced RAW264.7 cells via the Griess reaction. NO, which is synthesized by iNOS plays a key role in the pathogenesis of inflammation in abnormal situations. PLM showed potent inhibitory effects in a concentration-dependent manner on NO production. Therefore, it can be expected that PLM will relieve inflammation. Under inflammation, macrophages, one of the important components of the immune system, have important roles like the production of large amounts of IL-1β, IL-6, and TNF-α. PLM at more than 200 μg/mL suppressed the production of LPS-induced pro-inflammatory cytokines such as IL-1β, IL-6, and TNF-α (Fig. 2).

Proteose-peptone (PP) as a known powerful stimulator of macrophages has been used for the induction of acute inflammatory PMN in various reports [6, 25–27]. Moreover, Starch which can induce mucosal as well as systemic immune responses is a natural biocompatible and biodegradable polymer [28] Th1 cells are required primarily to combat intracellular pathogens such as bacteria, viruses, and parasites. Th1 type is characterized by the release of IFN-γ, IL-2, IL-12, and TNF-α which favor macrophage activation [29]. Therefore, in general, Th1 cells are associated with the development of cellular immunity. On the other hand, Th2 cells are required extracellular forms of pathogens, which do not amplify inside an antigen-presenting cell (APC) and thus are not involved in antigen presentation. The cytokines are IL-4, IL-10, IL-5, and IL-13, which inhibit macrophage activation, and are implicated in the production of IgE and activation of mast cells and eosinophils. Cytokines play a vital role in the modulation of an immune response by influencing whether a response against an acquired infection will be dominated by Th1 or Th2 [30]. Moreover, inflammatory cytokines strengthen the presentation of co-stimulatory molecules recognized by CD4^+^ or CD8^+^ cells [31]. In the present study, all drug-treated groups increased IL-2, IL-12, and IFN-γ production by splenocytes compared to PP and starch-stimulated mice (control) (Fig. 2A, B, and C). Especially, PLM200 treatment showed a superior increase than that of other concentrations in all of the three cytokines above. Moreover, the ratio of IFN-γ /IL-4 production was significantly higher than the control group and consistent with the previous paper [32]. Compared with the control group, PLM200 significantly increased the ratio of IFN-γ /IL-4 production by 39.14% (Fig. 2D). Our result was consistent with other experiments [33, 34]. Accordingly, these results strongly suggest that PLM is a potent immune-stimulating extract.

Humans may come into contact with various pathogenic organisms through many other ways including skin contact, ingestion, and inhalation [35]. Herein, the innate immune system plays an essential role in preventing infection by a specific pathogen. However, adaptive immune responses to a new pathogen are relatively slower than innate immune responses. We evaluated the total cell number of the thymus, PEC, and PBMC related to immunity (Fig. 3). All drug-treated groups increased, especially, both KRG100 and PLM200 appeared a significant elevation. PLM showed the elevation of 4.0-fold, 1.7-fold, and 1.5-fold in the thymus, PEC, and PBMC, respectively. These results are associated with the increase of cells which can exert immunomodulatory activity.

Usually, lymphocytes (T and B) are key players in the adaptive immune response, which exerts more effective immune responses [36, 37]. Furthermore, antigen receptors of each naive lymphocyte possess unique specificity. Interestingly, B cells led to the secretion of antibodies, known as humoral immunity, while T cells led to cell-mediated immunity through subdivision into T helper cells (CD4^+^, called Th) and cytotoxic T cells (CD8^+^, called Tc). When the expression of immune cell subtypes was performed using flow cytometry, the absolute number of CD4^+^CD25^+^ (markers for regulatory T cells, called Treg), CD4^+^&CD8^+^_,_ and CD4^+^CD69^+^ by PLM200 treatment significantly increased 6.1-fold, 3.4-fold, 3.2-fold, respectively compared with the control group (Fig. 4). Moreover, absolute number of CD4^+^&CD8^+^ and CD4^+^CD69^+^ in PBMC showed a significant elevation of 66.5% at CD4^+^ and 209.1% at CD4^+^CD69^+^ by PLM200 supplementation. Furthermore, PLM200 showed a significant elevation of 31.26% at only CD69^+^ in CD4^+^&CD69^+^ (CD69 that represents the initial activity of T cell) in PEC (Fig. 5) [38]. These results clearly indicate that PLM is potent as immune-boosting material because it caused the activation of T subtypes in the thymus, PBMC, and PEC.

## Conclusions

In conclusion, our results showed an immunomodulatory effect of PLM through the increase of INF-γ/IL4 ratio and the anti-inflammatory effect through the inhibition of inflammatory mediators. Therefore, our results demonstrate that PLM with immunostimulatory and anti-inflammatory effects will raise the possibility of a new generation of orally available therapeutic agents.

## Data Availability

The data are available from the corresponding author by appropriate request.

## Abbreviations

PLM: *Phellinus linteus* mycelium
LPS: Lipopolysaccharide
PEC: Peritoneal exudate cells
PBMC: Peripheral blood mononuclear cells
PP: Proteose peptone
NO: Nitric oxide
PGE2: Prostaglandin E2
TNF: Tumor necrosis factor
IL: Interleukin
IFN: Interferon
